# Smart Privacy Protection for Big Video Data Storage Based on Hierarchical Edge Computing

**DOI:** 10.3390/s20051517

**Published:** 2020-03-10

**Authors:** Di Xiao, Min Li, Hongying Zheng

**Affiliations:** College of Computer Science, Chongqing University, Chongqing 400044, Chinazhenghongy@cqu.edu.cn (H.Z.)

**Keywords:** smart privacy protection, hierarchical edge computing, color video, low computation complexity, cloud storage

## Abstract

Recently, the rapid development of the Internet of Things (IoT) has led to an increasing exponential growth of non-scalar data (e.g., images, videos). Local services are far from satisfying storage requirements, and the cloud computing fails to effectively support heterogeneous distributed IoT environments, such as wireless sensor network. To effectively provide smart privacy protection for video data storage, we take full advantage of three patterns (multi-access edge computing, cloudlets and fog computing) of edge computing to design the hierarchical edge computing architecture, and propose a low-complexity and high-secure scheme based on it. The video is divided into three parts and stored in completely different facilities. Specifically, the most significant bits of key frames are directly stored in local sensor devices while the least significant bits of key frames are encrypted and sent to the semi-trusted cloudlets. The non-key frame is compressed with the two-layer parallel compressive sensing and encrypted by the 2D logistic-skew tent map and then transmitted to the cloud. Simulation experiments and theoretical analysis demonstrate that our proposed scheme can not only provide smart privacy protection for big video data storage based on the hierarchical edge computing, but also avoid increasing additional computation burden and storage pressure.

## 1. Introduction

Cloud computing was introduced in Search Engine Strategies 2006 in order to tackle the constraints of the Internet of Things (IoT) in management, storage, computing and processing, and was formally defined by National Institute of Standards and Technology [1]. Since then, this prominent computing infrastructure has always drawn attentions of increased individuals, enterprises and researchers with its affordable prices, powerful communication facilities, abundant computing and storage resources [2]. These advantages make it possible to offer more services (e.g., cloud storage [3], smart monitoring and forecasting [4]) by integrating plentiful connected sensor devices.

However, cloud computing cannot efficiently support big multimedia data storage in distributed IoT environments because of the following reasons [5]. First, the mobile sensor application facilities require real-time response, location awareness and mobility, but cloud computing fails to satisfy these demands due to being far away from local sensor devices. Second, cloud computing processes huge amounts of raw data directly, which results in inevitable service delays and process blocking. Third, cloud services face substantial unprecedented challenges with exponential rise of user volume. In order to better address the above issues, edge computing [6,7], a new decentralized paradigm, was proposed to extend cloud computing to the edge of networks. This popular technology contains seven features, that is, dense geographical distribution, mobility support, location awareness, context-awareness, low latency, proximity and heterogeneity so that it can improve the efficiency and quality of cloud services and be suitable for strict and smart application scenarios [8].

**Figure 1:**
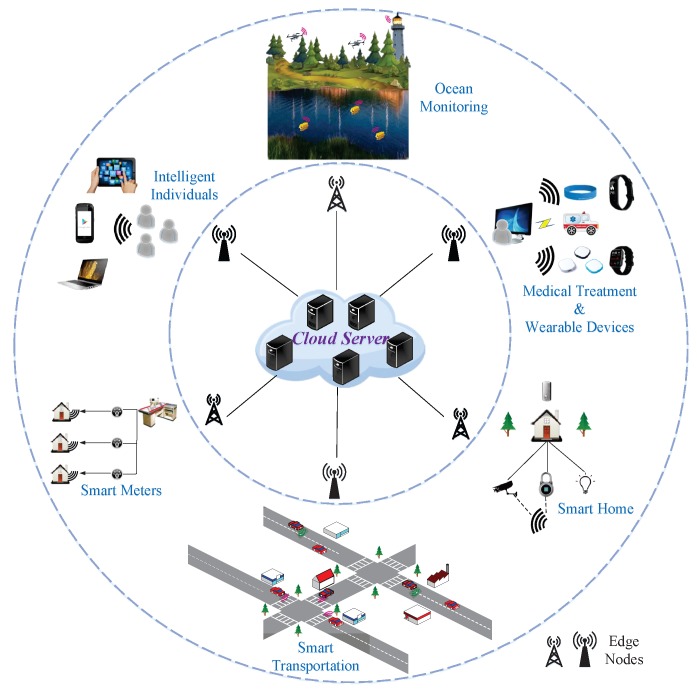
The overall architecture of edge computing.

Generally speaking, edge computing includes three patterns, that is, multi-access edge computing (MEC) [9,10], cloudlets [11] and fog computing [12]. To be brief, MEC is an emergency model that uses mobile base stations as sensor nodes to handle delay-sensitive and context-aware applications. Different from set-top-boxes, road-side units, routers, gateways and other resource-limited devices, cloudlets have powerful CPU and rich storage spaces. Fog computing is regarded as a scenario in which a large number of heterogeneous and decentralized base stations and access points act as fog nodes to communicate and cooperate with each other and perform information processing and storage operations in the absence of the third-party. The overall architecture of edge computing is shown in Figure 1. The outermost layer is the local facilities which are formed by plentiful mobile sensor devices such as smart transportation, medical treatment and wearable services. Original data can be generated or sampled here and transmitted to the middle layer for the next process. The middle layer is the edge layer which contains numerous edge nodes with computing and storage capabilities. It exists for the purpose of providing transient data storage and computing offloading for users and simultaneously alleviating the cloud burden. The central layer is the cloud server which supports further huge data processing and storage.

However, various security issues have always been potential threats to the cloud storage. Each user has his/her own data ranging from GB’s to TB’s and the local storage fails to achieve this huge demand alone. Thus, deploying a low-complexity and high-secure cloud storage service has become an inevitable trend. In other words, the cloud provider who supports smart edge computing-based IoT applications with higher security degrees will attract more individuals. In this paper, we propose a novel distributed compressive video sensing (DCVS) coding [13] and the hierarchical edge computing architecture-based scheme to offer a low-complexity and high-secure big video data storage service. Our main contributions can be summarized as follows.

- We make full use of the respective advantages of MEC, cloudlets and fog computing to design the hierarchical edge computing architecture to support distributed IoT application environments.
- The most significant bits (MSB) of the key frame (KF) are completely controlled by users so that our proposed scheme has higher degrees of security. Meanwhile, the least significant (LSB) bits of KF are directly encrypted via MSB to avoid unnecessary computation burden of edge computing and extra storage pressure of local sensor devices.
- The two-layer parallel compressive sensing is used to compress the non-key frame (NKF) so as to minimize the storage burden on cloud services.

The remainder of this paper is organized as follows. Section 2 briefly provides necessary and basic relevant knowledge. Section 3 describes the scheme of smart privacy protection for big video data storage based on the hierarchical edge computing in detail. Section 4 evaluates performance of the proposed scheme from both experiments and theory and Section 5 draws a brief conclusion.

## 2. Related Work

### 2.1. Bit Adaptive Diffusion

Diffusion can significantly change statistical characteristics of the input plain-frame and spread the influence of each bit to the cipher-frame. The principle is to diffuse the corresponding pixel of the current frame through the value of the key stream [14]. In order to achieve high communication efficiency of hardware in cloud computing, we adopt bit adaptive diffusion by bit-wise XOR operation. It can be defined as

(1) $$A_{i,j}=\left\{\begin{array}{c} O_{i,j}⊕O_{I,J}⊕S_{i,j},\;\;\;\;\;\;\;\;\;\;\;\;\;\;\;if\;\;\;i=1,j=1,\\ O_{i,j}⊕A_{I,j-1}⊕S_{i,j},\;\;\;\;\;\;if\;\;\;i=1,j\neq 1,\\ O_{i,j}⊕A_{i-1,j}⊕S_{i,j},\;\;\;\;\;\;\;\;\;\;\;\;\;\;\;\;\;\;\;\;\;\;\;\;\;\;\;\;\;\;\;\;\;\;\;if\;\;\;i\neq 1, \end{array}\right.$$

where ⊕ is the bit-wise XOR operation. *S* is the key stream with the same size $\left(I\times J\right)$ and data format as the original frame *O*. The inverse operation in the decryption process can be expressed by

(2) $$O_{i,j}=\left\{\begin{array}{c} A_{i,j}⊕O_{I,J}⊕S_{i,j},\;\;\;\;\;\;\;\;\;\;\;\;\;\;\;if\;\;\;i=1,j=1.\\ A_{i,j}⊕A_{I,j-1}⊕S_{i,j},\;\;\;\;\;\;if\;\;\;i=1,j\neq 1.\\ A_{i,j}⊕A_{i-1,j}⊕S_{i,j},\;\;\;\;\;\;\;\;\;\;\;\;\;\;\;\;\;\;\;\;\;\;\;\;\;\;\;\;\;\;\;\;\;\;\;if\;\;\;i\neq 1. \end{array}\right.$$

### 2.2. Parallel Compressive Sensing

Compressive sensing (CS) has two promising advantages: compress and sample simultaneously; the sampling rate is much lower than the Nyqvist-Shannon criterion. Both of them facilitate CS to be used in relevant fields widely since its emergence [15]. However, the multidimensional signal has to be transformed to the 1D signal before compression so that the required measurement matrix is tremendous. In order to reduce computation complexity and storage space, the characteristics of 2D signals are combined with the theory of CS to be parallel CS (PCS) [16] is suggested. The mathematical expression is as follows

(3) $$y_{i}=\Phi x_{i},$$

where $x_{i}$ is the *i* column of an original 2D signal and $y_{i}$ is the *i* column of the measurement value via linear projection of the measurement matrix Φ.

### 2.3. Researches on Relevant Privacy-Protection Schemes

Lyu et al. proposed a privacy-preservation system for fog-based aggregation in Smart Grid with differential privacy so as to enable the intermediate fog nodes to safely collect data from connected smart meters [17]. Wang et al. presented a three-layer privacy preserving cloud storage scheme in cloud computing to resist possible attacks from the inside of cloud servers [18]. Xue et al. designed verifiable security fine-grained access control in vehicular cloud computing to share latency-sensitive data [19]. Gu et al. provided a dynamic method to protect user privacy during communications between users and multiple fog nodes and meanwhile discussed the payoff and privacy loss in time of the process [20]. Wang et al. proposed an effective dual-chaining watermark scheme for data integrity protection in smart campus IoT applications, and this smart meteorological Internet of Things system can effectively authenticate the integrity of the data with free distortion at low cost [21]. Wang et al. proposed an edge-based model for data collection to provide a general privacy preservation service via the differential privacy algorithm [22]. Wang et al. designed a scheme that attempts to preserve a balance in user privacy, data integrity in edge-assisted IoT devices and the computational cost through a balanced truth discovery approach and a proposed enhanced technique [23]. He et al. proposed a distributed privacy preserving scheme for random linear network coding in smart grid that offers a data confidentiality privacy preserving feature and efficiently thwarts traffic analysis [24]. Xie et al. designed a new efficient privacy preserving compressive data gathering scheme, which exploits homomorphic encryption functions in compressive data gathering to thwart the traffic analysis/flow tracing and possess message flow un-traceability and message content confidentiality [25]. Gu et al. presented a scheme for the privacy protection of location data mining based on the differential privacy mechanism, which protects highly frequent accessing location data or location preference of user by distorting accessing frequencies [26]. It is worth affirming that the above researches have achieved great contributions of privacy protection based on the cloud computing in different aspects.

However, to the best of our knowledge, this study is the first to take full advantage of MEC, cloudlets and fog computing to design the hierarchical edge computing architecture and propose a smart and low-complexity privacy protection scheme for big video data storage based on it. Besides, the cloud space does not mean unlimited storage resources so that we also consider compression.

### 2.4. Researches on Relevant Edge Computing Schemes

Bonomi et al. argued that the features of fog computing make it suitable for many critical Internet of Things services and applications, namely, connected vehicle, smart grid, cloud computing and wireless sensors networks [27]. Hu et al. showed that MEC enables innovative service scenarios that can ensure enhanced personal experience and optimized network operation, and satisfy the demanding requirements for ultra-low latency and stimulating innovation [28]. A novel vision of mobile computing liberates mobile devices from severe resource constraints by enabling resource-intensive applications to use cloud computing free of jitter, congestion and failures [29]. Unfortunately, longer communication delay is a fundamental obstacle. Rather than relying on a distant “cloud”, it is better to address a mobile device’s resource poverty through the nearby resource-rich edge computing.

Mora-Gimeno et al. presented a security model for the externalization of application execution in multi-tier MEC environments, which produces a minimal overhead, especially for computationally intensive applications [30]. Lee et al. designed a hierarchical MEC architecture in which MEC servers are arranged in a hierarchical way to provide users with rapid content delivery, higher computing performance, and efficient use of server resources [31]. Dong et al. studied a dynamic and decentralized resource-allocation strategy based on the evolutionary game theory to deal with task offloading to multiple heterogeneous edge nodes and central clouds among multi-users, which could achieve one evolutionary equilibrium, and meet users’ quality-of-service requirements under resource constraints of edge nodes [32]. Lee et al. proposed the MEC-based mobility management scheme that arranges MEC server as the concept of Zone so that mobile users can continue to receive content and use server resources efficiently even when they move [33].

The above researches related with the edge computing schemes can be classified as the three-level architecture, including the local individual layer, the edge computing layer and the cloud computing layer. Specifically, the edge computing layer is refined as the MEC layer. However, although MEC brings computing power and storage resources to the edge of the mobile network, limited computation resources of mobile edge nodes may not be sufficient to serve excessive offloading tasks exceeding the computation capacities of themselves. Further, we can take advantage of the lower latency of MEC to distinguish KF from NKF in real time while receiving the video. Meanwhile, all frames are encrypted or compressed by fog computing, which has relatively stronger computing resources compared with MEC. In addition, considering the local storage resources are limited and individuals do not want the key information of the video to be stored by completely un-trusted cloud, we use the independent encoding - joint decoding DCVS coding to divide the video data into three parts. MSB of KF is stored by local devices while LSB of KF is transmitted to the semi-trusted cloudlets. The rest, NFK, which must be reconstructed better via referring to KF, should be stored to the cloud after a series of processing. This is why we design a novel hierarchical edge computing architecture to provide smart private protection for big video data storage with low latency.

## 3. Description of the Proposed Scheme

**Figure 2:**
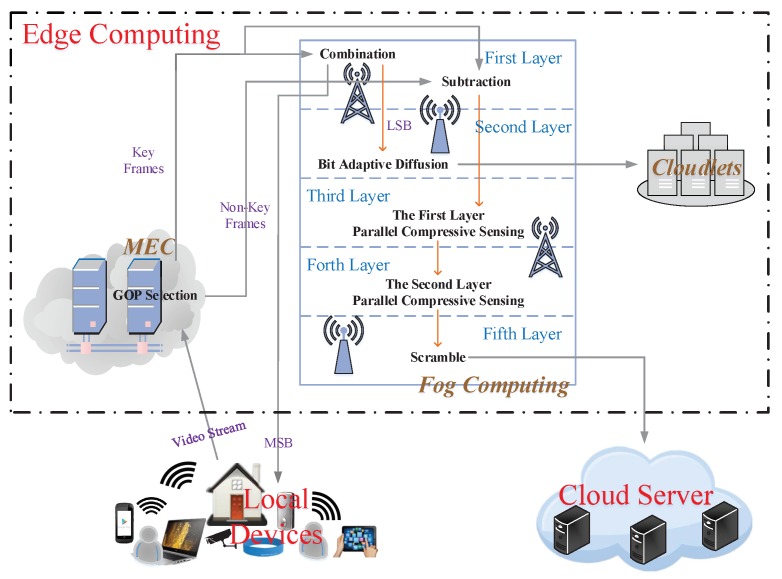
The overall scheme of privacy protection for big video data storage based on the hierarchical edge computing.

The overall scheme of using the hierarchical edge computing architecture to protect big video data storage privacy is shown in Figure 2. Firstly, local devices transmit video streams to the edge layer through wireless communication. Then, the raw videos are processed by the group of pictures (GOP) selection in MEC. The separated KF and NKF are encrypted and compressed respectively in fog computing. Significant and sensitive KF after processing are stored in local devices and cloudlets equally. The remainders are sent to the cloud server via wire communication. Both procedures and algorithms adopted in our proposed scheme will be described in detail as follows. It is worth noticing that we adopt color videos as objects to better fit the actual environment.

### 3.1. GOP Selection

Video is not a simple combination of pictures, and there are strong correlations among frames. That is, the selection of KF often determines the whole recovery performance of NKF from the same GOP. Thus, GOP selection has profound significance for video sampling. However, service objects of the cloud consist of intelligent users, ocean monitoring and so on, as shown in Figure 1, so we have to combine actual scenarios and computation costs to distinguish KF from NKF.

- Fixed GOP values: If a certain scenario needs to analyze complex or important videos, the small values should be selected to ensure reconstruction performance, while the large values could be considered to reduce the overall sampling rate of videos and handle massive data.
- Adaptive GOP selection: In order to improve encoding efficiency and decoding performance, the adaptive GOP selection based on the perceptual hash algorithm was applied for DCVS [34]. Though the computation complexity of this algorithm is relatively high, the accuracy of selecting KF is raised substantially.

In a word, we should make a concrete analysis of concrete conditions. MEC receives the video stream from the nearest local devices and then the separated KF and NKF are transmitted to different layers of fog computing.

### 3.2. Encoding and Decoding of Key Frames

**Figure 3:**
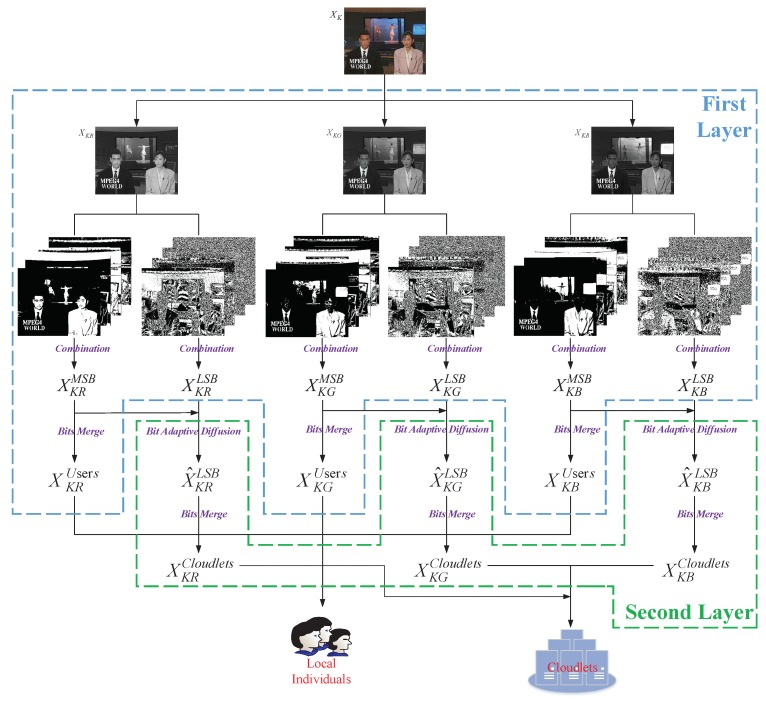
The encoding of key frame (KF) in fog computing.

Take 1st frame of “News(CIF)” as an example. The whole scheme of encoding KF is shown in Figure 3. The first layer of fog computing receives KF and separates the RGB three-layer of a color frame bit by bit all alone. These bit-layers can be denoted by $\left\{X_{Kj}^{i}\right\}$ where $i\in \left\{1,2,\ldots ,8\right\}$, $j\in \left\{R,G,B\right\}$, e.g., $X_{KR}^{1}$ is the first bit-layer of the R layer in the KF. As the operation of the RGB three-layer are the same so that we describe the R layer in particular.

The MSB $\left\{X_{KR}^{1},X_{KR}^{2},X_{KR}^{3},X_{KR}^{4}\right\}$ and LSB $\left\{X_{KR}^{5},X_{KR}^{6},X_{KR}^{7},X_{KR}^{8}\right\}$ of the original R layer are respectively combined to generate the 1D plane $X_{KR}^{MSB}$ and $X_{KR}^{LSB}$. Then $X_{KR}^{LSB}$ and the copied $X_{KR}^{MSB}$ are transmitted to the second layer of fog computing. Perform bit adaptive diffusion with $X_{KR}^{MSB}$ by

(4) $${\hat{X}}_{KR}^{LSB}\left(r.c\right)=\left\{\begin{array}{c} X_{KR}^{LSB}\left(r.c\right)⊕X_{KR}^{LSB}\left(4M,N\right)⊕X_{KR}^{MSB}\left(r,c\right),\;\;if\;\;\;r=1,c=1,\\ X_{KR}^{LSB}\left(r.c\right)⊕{\hat{X}}_{KR}^{LSB}\left(N,c\right)⊕X_{KR}^{MSB}\left(r,c\right),\;\;\;\;\;\;\;\;\;\;\;\;\;\;if\;\;\;r=1,c\neq 1,\\ X_{KR}^{LSB}\left(r.c\right)⊕{\hat{X}}_{KR}^{LSB}\left(r-1,c\right)⊕X_{KR}^{MSB}\left(r,c\right),\;\;\;\;\;\;\;\;\;\;\;\;\;\;\;\;\;\;\;\;\;\;\;\;\;\;\;\;\;if\;\;\;r\neq 1, \end{array}\right.$$

where $r\left(r\in \left[1,4M\right]\right)$ is the abscissa and $c\left(c\in \left[1,N\right]\right)$ is the ordinate. *M* and *N* are the width and the length of the KF $X_{K}$ respectively.

**Figure 4:**
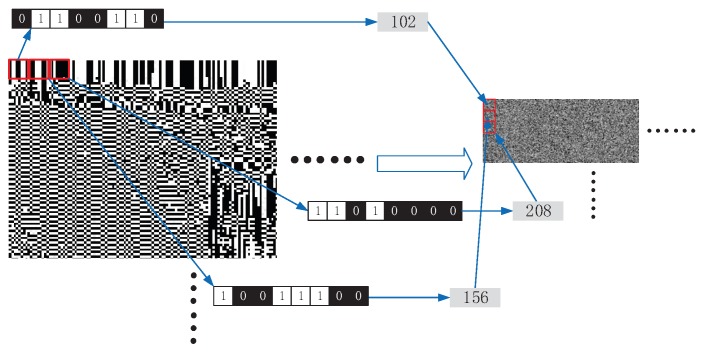
The visual representation of merging a binary matrix to a pixel matrix.

Then, the binary matrix $X_{KR}^{MSB}$ and the encrypted ${\hat{X}}_{KR}^{LSB}$ are merged bit by bit in the first layer and the second layer of fog computing respectively to obtain $X_{KR}^{Users}$ and ciphertext $X_{KR}^{Cloudlets}$. Specifically, the binary matrix merges each 8 bits into a new pixel as shown in Figure 4. Then send $X_{KR}^{Users}$ to local devices and send $X_{KR}^{Cloudlets}$ to cloudlets simultaneously.

As encoding of KF only involves encryption, decoding is the inverse process of encoding. In brief, $X_{KR}^{Users}$ and $X_{KR}^{Cloudlets}$ are obtained from local devices and cloud servers respectively at first, and then the original LSB are decrypted with the MSB binary stream. Finally, combine MSB with LSB to generate KF. Thus it can be seen KF, as the key information of the video, is further divided into two parts. The most important part of KF is directly stored in local devices, and meanwhile regarded as the key to encrypt the least important part. Considering the local storage resources are limited and individuals do not hope to transmit any data of KF to the un-trusted cloud, we could store LSB of KF with the help of cloudlets in order to obtain higher level of security.

### 3.3. Encoding and Decoding of Non-Key Frames

**Figure 5:**
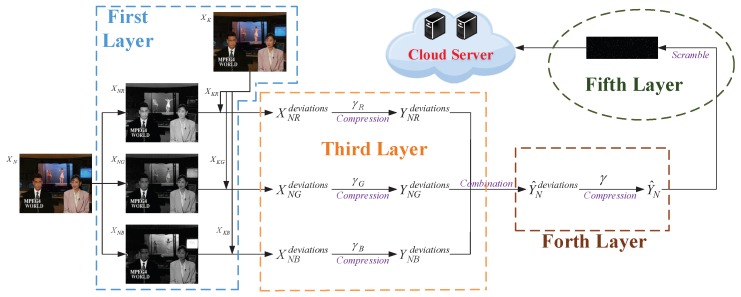
The encoding of non-key frame (NKF) in fog computing.

To further improve the security level and achieve better accuracy of the NKF reconstruction, we do not directly perform coding but extract deviations between NKF and KF of the same GOP at first in the first layer of fog computing. Then taking the 2nd frame of “News(CIF)” as an example, as shown in Figure 5, the process of the first layer PCS is to compress the RGB three-layer of deviations independently in the third layer of fog computing. It can be represented visually as follows

(5) $$\left\{\begin{array}{c} Y_{NR}^{deviations}=\Phi_{NR}\;X_{NR}^{deviations},\\ Y_{NG}^{deviations}=\Phi_{NG}\;X_{NG}^{deviations},\\ Y_{NB}^{deviations}=\Phi_{NB}\;X_{NB}^{deviations}, \end{array}\right.$$

where $\left\{X_{Nj}^{deviations}\left|j\in \left\{R,G,B\right\}\right.\right\}$ is the RGB three-layer of the original deviations between KF and NKF, and $\left\{Y_{Nj}^{deviations}\left|j\in \left\{R,G,B\right\}\right.\right\}$ is the corresponding measurement values. Let us discuss the generation of the measurement matrix $\left\{\Phi_{Nj}\left|j\in \left\{R,G,B\right\}\right.\right\}$. To ensure the better reconstruction performance and satisfy the easier implementation of hardware simultaneously, the deterministic binary block diagonal matrix (DBBD) [35] is adopted here. Moreover, considering the level of security, we select different sampling rates to compress each layer of deviations. Then convert $Y_{N}^{deviations}$ to a 1D plane ${\hat{Y}}_{N}^{deviations}$ and transmit it to the forth layer of fog computing. The process of the second layer PCS is to compress ${\hat{Y}}_{N}^{deviations}$ with $\Phi_{DBBD}$ to obtain ${\hat{Y}}_{N}$. For a certain NKF, the total sampling rate is

(6) $$\Gamma =\left[\left(\gamma_{R}+\gamma_{G}+\gamma_{B}\right)÷3\right]\times \gamma ,$$

where $\gamma_{R},\gamma_{G},\gamma_{B}$ denote the sampling rate of the RGB three-layer respectively and γ is that of ${\hat{Y}}_{N}^{deviations}$. Although we do not directly process the original NKF, the measurement matrix used to reduce computation complexity and storage pressure is not secure, so the information after compression needs to be further encrypted. Send ${\hat{Y}}_{N}$ to the fifth layer of fog computing and the steps of scramble are shown below.

1. Set initial values $\alpha_{0},\beta_{0}$ and a control parameter μ$\left(\alpha_{0},\beta_{0},\mu \in \left(0,1\right)\right)$.
2. Iterate H×W+l rounds by 2D logistic-skew tent map with $\alpha_{0},\beta_{0},\mu$ and discard first *l* iterated values to avoid transient effects by
  (7) $$\left\{\begin{array}{c} \alpha_{i+1}=4\times \beta_{i}\times \left(1-\beta_{i}\right),\;\;\;\;\;\;\;\;\;\;\;\;\;\;\;\;\;\;\;\;\;\;\;\;\;\;\;\;\;\;\;\;\;\;\;\;\;\;\;\;\;\;\;\;\;\;\;\;\;\;\;\\ \beta_{i+1}=\left\{\begin{array}{c} \alpha_{i}/\mu ,\;\;\;\;\;\;\;\;\;\;\;\;\;\;\;\;\;\;\;\;\;\;\;\;\;\;\;\;\;\;\;\;\;\;\;\;\;\;\;\;\;\;\;\;\;\;\;\;\;\;\;\;\;\;\;0<\alpha_{i}<\mu ,\\ \left(1-\alpha_{i}\right)/\left(1-\mu \right),\;\;\;\;\;\mu \leq \alpha_{i}<1, \end{array}\right. \end{array}\right.$$
  where H×W is the size of ${\hat{Y}}_{N}$.
3. Discretize α,β and obtain two sequences $\alpha^{\prime },\beta^{\prime }$ ($j\in \left[1,H\times W\right]$),
  (8) $$\left\{\begin{array}{c} {\alpha^{\prime }}_{j}=\left\lfloor \alpha_{j}\times 10^{14}\right\rfloor \;\;\;\;\;\;\mathrm{mod}\;H+1.\\ {\beta^{\prime }}_{j}=\left\lfloor \beta_{j}\times 10^{14}\right\rfloor \;\;\;\;\;\;\mathrm{mod}\;W+1. \end{array}\right.$$
4. Rearrange $\alpha^{\prime },\beta^{\prime }$ into two matrices with the size $\left[H,W\right]$ respectively.
5. Exchange the pixel ${\hat{Y}}_{N}\left(h,w\right)$ and ${\hat{Y}}_{N}\left({\alpha^{\prime }}_{h,w},{\beta^{\prime }}_{h,w}\right)$ from the top left corner to the bottom right corner in order where $h\in \left[1,H\right]$, $w\in \left[1,W\right]$, $\alpha_{h,w}^{\prime }$ and $\beta_{h,w}^{\prime }$ are values in matrices $\alpha^{\prime },\beta^{\prime }$ respectively.

Finally, transmit the encrypted NKF to the cloud.

The decoding process of NKF is decryption-then-reconstruction. Decryption and encryption are inter-reversible and the secret key $\alpha_{0},\beta_{0},\mu$ must be unified. The total variation minimization by augmented Lagrangian and alternating direction algorithm (TVAL3) [36] is used for reconstruction. At last, the recovered deviations plus the decrypted KF can obtain the corresponding decoded NKF.

## 4. Simulation Results and Performance Evaluations

### 4.1. Experimental Results

**Figure 6:**
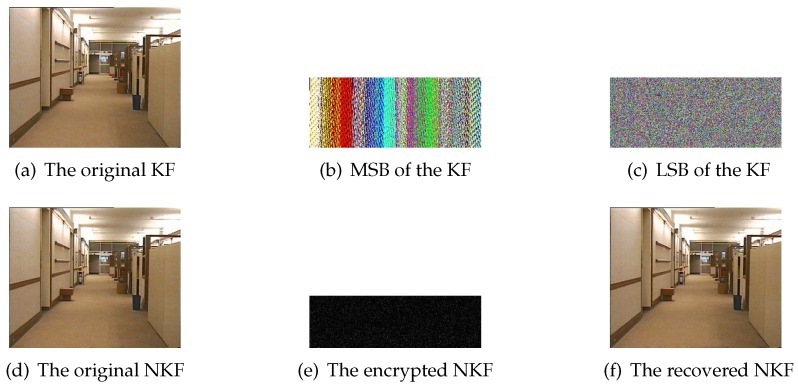
The simulation results of Hall(CIF).

**Figure 7:**
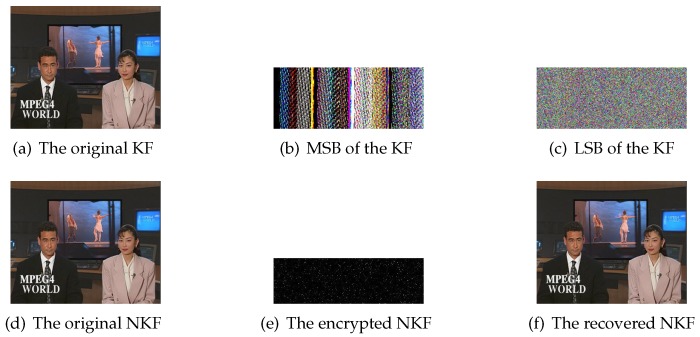
The simulation results of News(CIF).

**Figure 8:**
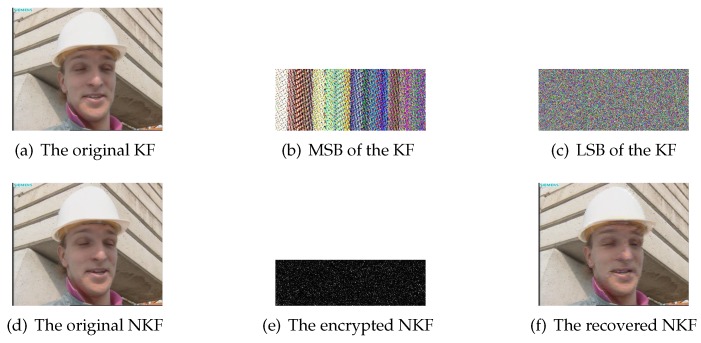
The simulation results of Foreman(CIF).

In this paper, our proposed scheme is encoded and decoded by MATLAB 2016a programming software. Hall(CIF), News(CIF) and Foreman(CIF) with different motion types are applied to simulate our scheme reliably. Figure 6a,d show the original 1st KF and 2nd NKF of Hall(CIF), respectively. Figure 6b denotes MSB of the KF and Figure 6c is LSB of that. Figure 6e is the encrypted NKF. The first-layer PCS adopts $\gamma_{R}=0.3$, $\gamma_{G}=0.25$ and $\gamma_{B}=0.2$ while the second-layer PCS employs γ=0.5. Figure 6f is the reconstructed NKF and the peak signal to noise ratio (PSNR) value reaches up to 35.68. It can be seen that the reconstruction performance is satisfying with the overall sampling rate 0.125. Similarity, Figure 7 represents simulation results of KF and NKF in the first GOP of News(CIF), and Figure 8 shows simulation results of KF and NKF in the first GOP of Foreman(CIF).

### 4.2. Theoretical Security Analysis

The security analysis of video data storage based on the hierarchical edge computing can be analyzed from two aspects. From the perspective of KF, although the local individuals have full control over the edge computing devices and can rely on the cloudlets to manage data, cipher-data are stored to prevent an attacker who obtains the data by force. In the field of reversible data hiding, the LSB layers of the cover image are usually replaced by significant information to ensure that no change in the cover image is visible to the naked eye. It indicates that the important data of an image is located in the MSB layers. Based on this principle, we divide KF into the MSB layers and LSB layers equally. Compared with the random matrix generated by the key to diffuse the LSB layers, directly regarding the MSB layers as the key stream is far more secure and even does not need to provide the extra space to store the key. Besides, two secret keys with only one bit difference can encrypt a certain frame into two different cipher-frames by bit adaptive diffusion. So our secure scheme for KF has strong capability of resisting famous attacks, such as differential attack and chosen-plaintext attack.

The NKF, which occupies a large part of videos, have to be stored in the completely untrusted public cloud. So the privacy and compression should be considered at the same time. In order to achieve the above goal without increasing computation complexity, we directly adopt the two-layer PCS with controlled sampling rates. To be more specific, in the first compression process, the three-layer RGB employs different sampling rates so that the correct composition proportion of the 1D plane formed by combination could not be known absolutely. To further reduce the storage, the second compression is adopted. Besides, instead of processing NKF directly, the main information is removed by subtraction in order to avoid the sensitive data leakage. We also use 2D logistic-skew tent map to encrypt measurement values further to improve the performance of privacy preservation.

In short, our proposed scheme not only provides effective and efficient privacy protection for big video storage based on the hierarchical edge computing, but also does not add additional burden from both computation and storage aspects of edge computing and the public cloud.

### 4.3. Computation Complexity

Table 1 shows that the average encoding time required for MSB is 0.106 s and for LSB is 0.841 s of any KF in News(CIF). As part encoding stages of MSB and LSB can be carried out parallelly, the total encoding time of KF is generally less than 0.7 s. Besides, the average decoding time of KF is about 1 s. For NKF, as shown in Table 2 and Table 3, the average run-time of the whole encoding is only 0.113 s, but the decoding time is long due to higher iterative complexity of CS reconstruction. But the performance of existing non-iterative reconstruction algorithm can be optimized to greatly reduce the run-time. Most importantly, all frames of the video stream can be encoded simultaneously, that is to say, the total encoding time of the video stream is no more than 1 s in our proposed scheme, which indicates that our scheme has higher efficiency and low complexity.

### 4.4. Comparison

Table 4 shows the performance comparison among our proposed scheme and other relevant ones. It can be clearly seen that the proposed scheme has its unique merits.

## 5. Conclusions

In this paper, we take full advantage of three patterns (MEC, cloudlets and fog computing) of edge computing to design the hierarchical edge computing. Based on this architecture, we further propose a novel smart privacy preservation scheme for big video data storage. For KF, MSB are stored directly in local sensor devices while LSB are encrypted by MSB and then sent to cloudlets. For NKF, two-layer PCS and encryption are performed at first, and finally transmitted to the cloud service. Experimental results and theoretical analysis prove that our proposed scheme is effective, efficient and lower-complexity, which provides smart privacy-protection for video contents. 

## Figures and Tables

**Table 1:** Average run-time (s/frame) performance of KF in our proposed scheme.

	Encoding	Decoding
MSB	0.106	0.123
LSB	0.841	0.905

**Table 2:** Average run-time (s/frame) performance of encoding NKF in our proposed scheme.

The First PCS	The Second PCS	Encryption
0.017	0.035	0.061

**Table 3:** Average run-time (s/frame) performance of decoding NKF in our proposed scheme.

Decryption	The First Reconstruction	The Second Reconstruction
0.05	29.09	31.72

**Table 4:** Compared with the relevant privacy protection schemes.

Schemes	Produce Redundant Data	Compression	Complexity	Application Scenarios
Ours	No	Yes	Low	Cloud storage
[17]	No	No	Medium	Date aggregation
[18]	Yes	No	Low	Cloud storage
